# Inspiratory-Activated Airway Vagal Preganglionic Neurones Excited by Thyrotropin-Releasing Hormone via Multiple Mechanisms in Neonatal Rats

**DOI:** 10.3389/fphys.2018.00881

**Published:** 2018-07-17

**Authors:** Lili Hou, Min Zhang, Xingyi Zhang, Zhenwei Liu, Pengyu Zhang, Dongying Qiu, Lei Zhu, Xin Zhou

**Affiliations:** ^1^Department of Respiratory and Critical Care Medicine, Shanghai General Hospital, Shanghai Jiao Tong University, Shanghai, China; ^2^Department of Neurobiology, School of Basic Medical Sciences, Fudan University, Shanghai, China; ^3^Department of Respiratory Medicine, Zhongshan Hospital, Fudan University, Shanghai, China; ^4^Department of Physiology and Pathophysiology, School of Basic Medical Sciences, Fudan University, Shanghai, China; ^5^Department of Gerontology, Zhongshan Hospital, Fudan University, Shanghai, China

**Keywords:** thyrotropin-releasing hormone, airway vagal preganglionic neuron, gap junction, oscillation, patch clamp, asthma

## Abstract

The airway vagal preganglionic neurons (AVPNs) providing projections to intrinsic tracheobronchial ganglia are considered to be crucial to modulation of airway resistance in physiological and pathological states. AVPNs classified into inspiratory-activated AVPNs (IA-AVPNs) and inspiratory-inhibited AVPNs (II-AVPNs) are regulated by thyrotropin-releasing hormone (TRH)-containing terminals. TRH causes a direct excitatory current and attenuates the phasic inspiratory glycinergic inputs in II-AVPNs, however, whether and how TRH influences IA-AVPNs remains unknown. In current study, TRH regulation of IA-AVPNs and its mechanisms involved were investigated. Using retrogradely fluorescent labeling method and electrophysiology techniques to identify IA-AVPNs in brainstem slices with rhythmic inspiratory hypoglossal bursts recorded by a suction electrode, the modulation of TRH was observed with patch-clamp technique. The findings demonstrate that under voltage clamp configuration, TRH (100 nM) caused a slow excitatory inward current, augmented the excitatory synaptic inputs, progressively suppressed the inhibitory synaptic inputs and elicited a distinctive electrical oscillatory pattern (OP). Such a current and an OP was independent of presynaptic inputs. Carbenoxolone (100 μM), a widely used gap junction inhibitor, fully suppressed the OP with persistence of TRH-induced excitatory slow inward current and augment of the excitatory synaptic inputs. Both tetrodotoxin (1 μM) and riluzole (20 μM) functioned to block the majority of the slow excitatory inward current and prevent the OP, respectively. Under current clamp recording, TRH caused a slowly developing depolarization and continuously progressive oscillatory firing pattern sensitive to TTX. TRH increased the firing frequency in response to injection of a square-wave current. The results suggest that TRH excited IA-AVPNs via the following multiple mechanisms: (1) TRH enhances the excitatory and depresses the inhibitory inputs; (2) TRH induces an excitatory postsynaptic slow inward current; (3) TRH evokes a distinctive OP mediated by gap junction.

## Introduction

Bronchial asthma, a type of common chronic airway disease worldwide, has prominent features of airway hyper-responsiveness, inflammation and excessive activation of cholinergic fibers to the trachea and bronchioles. The mechanisms of neural reflex pathways involved are regarded as very primary points in the process of this disease. In reflex pathways the AVPNs located in medulla oblongata conveying signals from brain to the intrinsic tracheobronchial ganglia are considered to be very crucial for regulating airway function either in diseased conditions or normal states (Haxhiu et al., 2005; McGovern and Mazzone, 2010).

Airway vagal preganglionic neurons have been mainly found in the external compact of nucleus ambiguus (eNA) via application of fluorescent tracer to the extrathoracic tracheal wall (Haselton et al., 1992; Haxhiu and Loewy, 1996; Kc et al., 2004; Chen Y. et al., 2007; Qiu et al., 2011; Chen et al., 2012a,b; Hou et al., 2012, 2015; Zhou et al., 2013; Ge et al., 2015; Guo et al., 2017; Yan et al., 2017). AVPNs in the eNA function as the main neurons which modulate cholinergic tone of airway smooth muscles as well as control the intrapulmonary airway resistance and tracheobronchial caliber (Haselton et al., 1992; Canning and Fischer, 2001; Yan et al., 2017). As shown in previous studies *in vivo* and *in vitro*, postganglionic neurons in intrinsic tracheobronchial ganglia have been identified as “phasic” neurons and “tonic” ones, the former firing in phase with inspiration and primarily projecting to tracheobronchial smooth muscles, and the latter firing tonically during expiration and primarily projecting to the intercartilaginous spaces (Baker, 1986; Mitchell et al., 1987; Myers et al., 1990; Myers, 1998). AVPNs in the eNA also exhibit different rhythmic changes of synaptic inputs in parallel with central inspiratory activities (Chen Y. et al., 2007; Qiu et al., 2011; Chen et al., 2012a,b; Hou et al., 2012, 2015; Zhou et al., 2013; Ge et al., 2015; Guo et al., 2017; Yan et al., 2017). Thus, two types of AVPNs are identified according to their different synaptic control during inspiratory phase: some neurons activated by phasic excitatory inputs are identified as IA-AVPNs, and others inhibited by phasic inhibitory inputs are separated as inspiratory-inhibited airway vagal preganglionic neurons (II-AVPNs) (Chen Y. et al., 2007; Qiu et al., 2011; Chen et al., 2012a,b; Hou et al., 2012; Zhou et al., 2013; Ge et al., 2015; Guo et al., 2017; Yan et al., 2017). Morphological and electrical studies have shown that excitatory and inhibitory synaptic inputs from various brain regions determine the excitability of AVPNs (Haxhiu et al., 1993, 2002, 2003, 2005, 2008; Kc et al., 2006; Wilson et al., 2007; Chen et al., 2012b; Hou et al., 2012; Zhou et al., 2013; Ge et al., 2015; Guo et al., 2017; Yan et al., 2017), although AVPNs are capable of generating synchronous electrical oscillatory pattern (OP), revealed by “activation of NMDA receptors” or “blockade of GABA_A_ receptors” (Haxhiu et al., 1987, 2005; Moore et al., 2004).

Nerves containing TRH innervate the airway vagal motor neurons in the cNA (Iwase et al., 1992; Sun et al., 1995). Microinjection of TRH, a neuropeptide well-known to excite the respiratory neurons in the PBC, into the NA induces first decrease and then increase of the tracheal pressure (Iwase et al., 1992). TRH affects neurons in the NA of adult guinea pigs by three different ways: depolarization, causing membrane potential oscillations and enhancement of postinhibitory rebound (Johnson and Getting, 1992), indicating that TRH could mediate complex alternations of membrane characteristics in neurons of NA. As demonstrated by the previous study in our laboratory, II-AVPNs were excited by TRH via both postsynaptic and presynaptic mechanisms (Hou et al., 2012). It has been reported that IA-AVPNs differ from II-AVPNs in anatomical location and intrinsic properties (Chen Y. et al., 2007; Chen et al., 2012a). Thus TRH modulation of IA-AVPNs is perhaps distinct from that of II-AVPNs, so how TRH regulates IA-AVPNs still remains elusive. In the current study, IA-AVPNs in the eNA were retrogradely labeled using fluorescent dye and identified in rhythmically active brainstem slices. The influences of TRH on whole-cell current and membrane potential of IA-AVPNs were examined using patch clamp. The current experiment is performed to test the following hypothesis that TRH affects IA-AVPNs via multiple mechanisms in neonatal rats: (1) TRH enhances the excitatory and inhibits the inhibitory synaptic inputs; (2) TRH induces a direct postsynaptic slow inward current; (3) TRH evokes a distinctive OP mediated by gap junction.

## Materials and Methods

### Ethical Approval

The experiments were performed on 120 newborn rats. The protocol was approved by the Ethical Committee of Shanghai Medical College affiliated to Fudan University (No. 20110307-060) and by the Animal Care Committee of Shanghai General Hospital affiliated to Shanghai Jiao Tong University, the research was carried out in line with the guideline of ”Guide for the Care and Use of Laboratory Animals” established by the National Institutes of Health.

### Retrograde Fluorescent Labeling of AVPNs and Preparation of Brainstem Slices

The 3- to 4-day-old Sprague-Dawley rats (Shanghai Institute for Family Planning and Shanghai General Hospital) were anesthetized with inhalation of halothane (0.5 ml) and hypothermia, and then the AVPNs were labeled retrogradely by rhodamine which have been described in details in our previous studies (Chen Y. et al., 2007; Chen et al., 2012a; Hou et al., 2012). Briefly, the extra thoracic trachea was exposed via a ventral midline incision in the neck. The fluorescent tracer rhodamine (XRITC, Molecular Probes, 1% solution, 0.5 μl) was injected into the tracheal wall between the fourth and eighth tracheal cartilage using a glass pipette of which the tip diameter was 30 μm with a syringe through polyethylene tubing attached. 48 h later, the animal was recovered. Halothane was used again to anesthetize the animal deeply as described above and then was decapitated. The brainstem was dissected out under a dissection microscope and then was submerged into ice-cold (4°C) ACSF which included (in mM) NaCl 124, KCl 3.0, KH_2_PO_4_ 1.2, CaCl_2_ 2.4, MgSO_4_ 1.3, NaHCO_3_ 26, D-glucose 10. The solution was constantly bubbled with 95% O_2_–5% CO_2_ to keep a pH of 7.4. The final osmolarity of ACSF was adjusted to 320 mosm/L with sucrose. The brainstem was then fixed with a chuck and submerged with the same ACSF in the slicing chamber. The rostral end of the brainstem was set upward; the dorsal surface was glued to an agar block facing the razor. Transverse brainstem slices of 500–800 μm thickness with two hypoglossal rootlets in each side (Smith et al., 1991) were cut using a vibratome (Leica VT 1000S). The brainstem slice with rhythmic inspiratory bursts (Smith et al., 1991) was transferred into the recording chamber (0.6 ml volume) to be superfused with high potassium (10 mM) ACSF at 23 ± 0.5°C. The rostral cutting plane of the slice was set upward to identify fluorescently labeled neurons and record synaptic activities of IA-AVPNs by patch clamp. The flow rate was maintained at 8–11 ml/min.

### Electrophysiological Recording

The AVPNs were first identified by their characteristic distribution in the eNA (Chen Y. et al., 2007; Chen et al., 2012a) and by presence of fluorescence with a 40×-water immersion objective lens. The patch pipettes which was filled with K^+^ gluconate-dominated internal solution included (in mM): K^+^ gluconate 150, HEPES 10, EGTA 10, CaCl_2_ 1, and MgCl_2_ 1; pH 7.3; 320 mosm/L. The holding voltage was normally set as −80 mV. In these recording conditions, only excitatory glutamatergic synaptic events were detectable, and the inhibitory synaptic currents mediated by chloride ion were minimized. When the IA-AVPN was clamped at −50 mV, both excitatory synaptic events manifesting inward currents and inhibitory synaptic events showing outward currents were detected. In some experiments, QX314 (lidocaine *N*-ethyl bromide, 2.0 mM) was put into the pipette solution to prevent activation of voltage-dependent sodium currents and slow inward rectifier (*I*_h_) (Marchetti et al., 2003; Sharifullina et al., 2005) of the patched neuron. IA-AVPNs were defined as those that were inspiratorily activated, as manifested by the rhythmic inspiratory discharges under cell-attached configuration (holding voltage 0 mV), by the rhythmic inspiratory bursts of the EPSCs under voltage clamp, or by the rhythmic inspiratory depolarizing EPSPs with superimposed trains of action potentials under current clamp (Chen Y. et al., 2007; Chen et al., 2012a). In each slice experiment was performed only on one IA-AVPN.

To get better recording of spontaneous inhibitory postsynaptic currents (sIPSCs) in some experiments, KCl-dominated internal solution was put into the patch pipette (in mM: KCL, 150; HEPES, 10; EGTA, 2; ATP-Mg, 2; pH 7.3; 320 mosm/L). The patched AVPNs were all clamped at −80 mV. Under this holding potential, both glutamatergic excitatory synaptic currents and sIPSCs in IA-AVPNs as well as sIPSCs in II-AVPNs were recorded as inward currents. In order to separate IA-AVPNs from II-AVPNs, the antagonists of glutamate receptors (CNQX and AP5) were topically applied to the patched neurons with the PV830 Pneumatic Picopump pressure delivery system (World Precision Instruments, Sarasota, FL, United States). IA-AVPNs were identified as those whose inspiratory inward currents could be abolished reversibly by CNQX and AP5.

The patch-clamp signal was amplified with an Axopatch 700B amplifier (10 kHz sampling frequency; 3 kHz filter frequency), digitized with 1322A digidata, and collected with Clampex 9.2 software (Axon Instruments, United States). The inspiratory hypoglossal bursts were recorded from hypoglossal rootlets using a suction electrode, amplified with a BMA-931 bioamplifier (5 kHz sampling frequency; 10–1,000 HZ band pass; 20,000 times), and electronically integrated (*τ* = 200 ms) with an MA-1000 Moving Averager (CWE Inc., Ardmore, PA, United States) before recording in the computer.

### Drug Application

Carbenoxolone and glibenclamide were dissolved in DMSO to make fresh stock solution of 100 mM and diluted to 100 μM in the bath to block gap junctions and inhibit ATP-sensitive potassium channels (K_ATP_), respectively. TRH affects the neural activity of inspiratory neurons and increases the discharging frequency of hypoglossal nerves in newborn mouse brainstem slices at the concentration of 1–5 μM (Rekling et al., 1996). In nucleus ambiguus neurons, 100 nM TRH induced membrane potential oscillations (Johnson and Getting, 1992). Thus two concentrations of TRH (1 μM and 100 nM) were used in this study at first. Because there were no significant differences between the effects of TRH on IA-AVPNs at these two concentrations, 100 nM was then used in this study. TRH was applied normally in the bath at 100 nM for 3–5 min. Strychnine (1 μM) and picrotoxin (40 μM) were used to block glycine receptors and GABA_A_ (γ-aminobutyric acid) receptors, respectively. CNQX (50 μM) and D-2-amino-5-phosphonovalerate (AP_5_; 50 μM) were used to block non-NMDA and NMDA-type glutamate receptors, respectively. When KCL-dominated internal solution was used to record synaptic currents, CNQX and AP5 were first topically applied to distinguish IA-AVPNs from II-AVPNs, and then were added into the perfusate to block EPSCs. In some experiments, TTX (1 μM) was included in the bath to prevent action potential generation and polysynaptic effects; riluzole (20 μM), to block persistent sodium currents (INaP). ACSF flowing into the chamber was all fresh and was not recycled. The drugs were purchased from Sigma-Aldrich (St. Louis, MO, United States).

### Data Analysis

The hypoglossal bursts and the TRH-evoked fast oscillatory currents (FOCs) in IA-AVPNs were analyzed with Clampfit 9.2 (Axon Instrument, United States). Spontaneous or miniature synaptic currents, as well as the ICSs phase-locked to the rapid inward phase of FOCs, were analyzed with MiniAnalysis (version 4.3.1, Synaptosoft), with a minimally acceptable amplitude at 10 pA. Regression analysis was performed with Origin 8.0 (OriginLab Corporation, Northampton, MA, United States). The results were presented as means ± SEM, and statistically compared with paired or independent Student’s *t*-test when appropriate. The significant difference was set at *P <* 0.05.

## Results

### Identification of Inspiratory-Activated Airway Vagal Preganglionic Neurons (IA-AVPNs)

**FIGURE 1:**
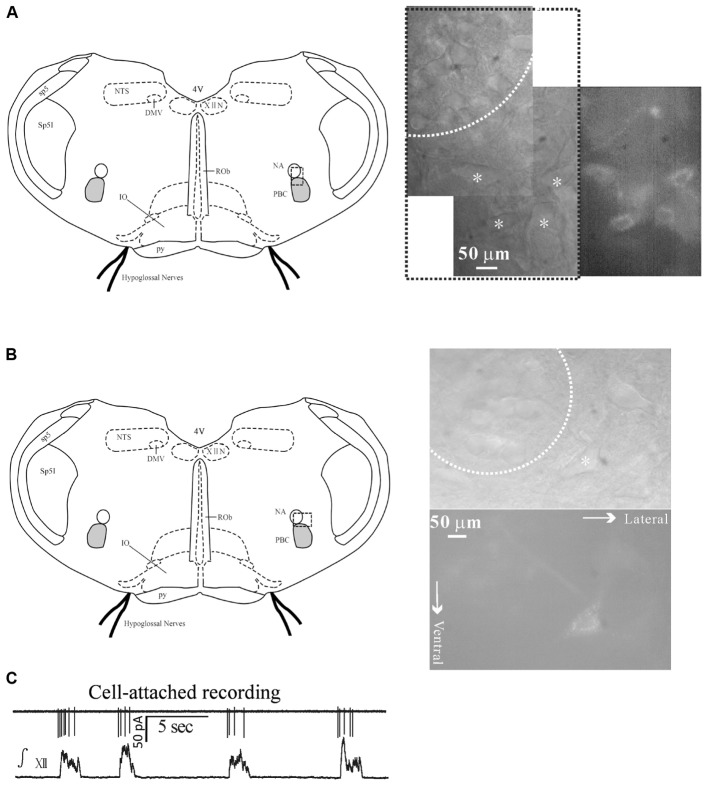
Identification of inspiratory-activated airway vagal preganglionic neurons (IA-AVPNs) in the external formation of NA. **(A,B)** After application of rhodamine into extra-thoracic tracheal wall fluorescently labeled IA-AVPNs (marked by ^∗^) in the ventrolateral medulla were mostly in the ventrolateral, and occasionally in the ventral or ventromedial vicinity (not shown) of the compact portion (dashed circle) of the nucleus ambiguus (NA). Note these cells were larger in size compared with those within the compact portion of the NA, and were typically multipolar or spindle-like. The dashed frames in the schematic figures of the medullary slices indicate the areas from which photos were taken under infrared or fluorescent illumination. **(C)** IA-AVPNs in the ventrolateral vicinity of the NA mostly exhibited trains of inspiratory-related discharges under cell-attached configuration and were identified as inspiratory IA-AVPNs. ∫ XII, integrated hypoglossal activity; XIIN, hypoglossal nucleus; NTS, nucleus tractus solitarius; DMV, dorsal motor nucleus of vagus; sp5, spinal trigeminal tract; Sp5I, spinal trigeminal nucleus, interpolar part; PBC, pre-Bötzinger complex; ROb, raphe obscurus nucleus; py, pyramidal tract; 4V, 4th ventricle.

Inspiratory-activated airway vagal preganglionic neurons were first identified by the presence of fluorescence and by their characteristic distribution in the eNA, which is in the close ventral, ventrolateral and ventromedial vicinity of the cNA (Chen Y. et al., 2007; Chen et al., 2012a) (**Figures 1A,B**).

**FIGURE 2:**
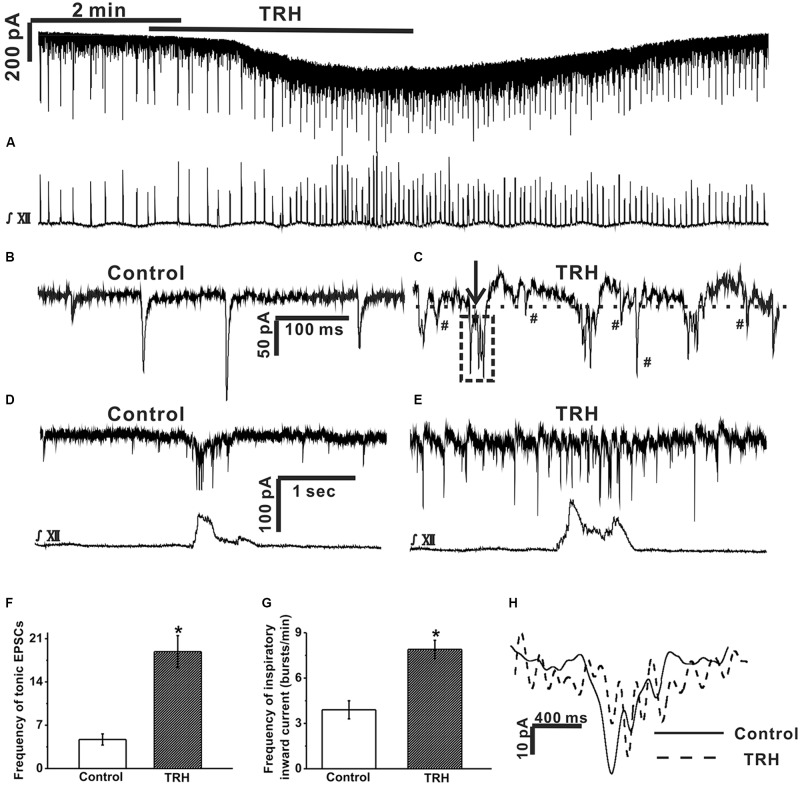
TRH caused an excitatory slow inward current and fast oscillatory currents in the inspiratory-activated airway vagal preganglionic neurons (IA-AVPNs). **(A)** Simultaneous recording of an IA-AVPN under voltage clamp (upper panel) and the hypoglossal respiratory bursts (lower panel), showing that TRH application caused a slow excitatory inward current in this IA-AVPN and increased the frequency and the intensity of the hypoglossal respiratory bursts. **(B,C)** Selected traces of the tonic EPSCs captured from **(A)** during control **(B)** and during TRH application **(C)** are shown in an enlarged scale. The dashed horizontal line in **(C)** indicates the range of the slower outward phase and the arrow shows a typical rapid inward phase of FOCs. The tonic EPSCs show a rhythmic-like change (the box in dashed line) synchronous with the FOCs during TRH application **(C)**. EPSCs (marked by #) occurred during the slower outward phase. The scale bars in **(B)** also applies to **(C)**. **(D,E)** The phasic EPSCs during control **(D)** and TRH-induced FOCs in a different IA-AVPN were also identifiable during inspiratory phase **(E)** with a shorter cycle length. The scale bars in **(D)** also applies to **(E)**. **(F)** Summarized data for the frequency of tonic EPSCs in average (*n* = 12). **(G)** Summarized data for the frequency of phasic inspiratory inward currents in average (*n* = 12). **(H)** Comparison of bell-shaped inspiratory inward currents in **(D)** (during control) and in **(E)** (during application of TRH). ^∗^*P* < 0.05, Student’s paired *t*-test.

Inspiratory-activated airway vagal preganglionic neurons were defined as those that were activated during inspiratory phase and were manifested by the rhythmic inspiratory-related discharges under cell-attached configuration (**Figure 1C**), by the rhythmic bursts of EPSCs during inspiratory phase under voltage clamp recording (**Figures 2A,D**) or by the rhythmic inspiratory-related depolarizing EPSPs superimposed by trains of action potentials under current clamp recording data not shown. Inspiratory inhibited AVPNs (II-AVPNs) were further defined as those that were inspiratorily inhibited and were manifested by the rhythmic bursts of the inhibitory (outward) postsynaptic currents (IPSCs) during inspiratory phase at holding voltages more positive than -50 mV or by the rhythmic hyperpolarizing inhibitory postsynaptic potentials (IPSPs) during inspiratory phase under current clamp recording (Chen Y. et al., 2007; Chen et al., 2012a). IA-AVPNs were mostly found in the close ventrolateral vicinity and II-AVPNs were mostly found in the near ventral or ventral medial vicinity of the cNA. From 120 slices, a total of 136 AVPNs were identified and tested, of which 120 were IA-AVPNs (88.2%) and 16 were II-AVPNs (11.8%).

### TRH Caused an Excitatory Slow Inward Current and Fast Oscillatory Currents (FOCs) in IA-AVPNs

Under voltage clamp, TRH (100 nM) increased baseline current noise and caused a slowly developing excitatory inward current (**Figure 2A**), which peaked within 3 min, with an average amplitude of 55.3 ± 19.7 pA (from 12 neurones). As the excitatory slow inward current developed, the baseline current generated a fast oscillatory change with respect to a steady level reached by TRH. This change was readily recognizable during the inspiratory intervals in all the 12 IA-AVPNs examined from 12 slices (**Figures 2B,C**), with an average cycle length of 201.2 ± 18.5 ms. In some (eight of twelve) IA-AVPNs, the change was also identifiable during the inspiratory phase (**Figures 2D,E**), with a significantly shorter cycle length of 90.1 ± 6.2 ms (*P* < 0.05 compared with the value during inter-inspiratory intervals; *n* = 8). The amplitude of this FOCs was relatively consistent in individual neurones, with an average of 38.3 ± 4.1 pA (*n* = 12). Each oscillatory cycle was composed of a rapid inward phase and a slower outward phase (**Figure 2C**). The rapid inward phase of each oscillatory cycle was superimposed by a bursting of EPSCs (**Figure 2C**), and the slower outward phase was superimposed by sporadically occurring EPSCs that were either relatively more frequent (**Figure 2C**) or very scarce (data not shown) in individual neurons. During TRH application the tonic EPSCs increased in the frequency (4.7 ± 0.9 HZ vs. 18.9 ± 2.6 HZ, *P* < 0.05, *n* = 12, Student’s paired *t*-test, **Figures 2C,F**) and showed a rhythmic-like change synchronized with FOCs (**Figure 2C**). TRH increased the frequency of the inspiratory inward currents from 3.9 ± 0.6 to 7.6 ± 0.9 bursts/min (*P* < 0.05, *n* = 12, Student’s paired *t*-test, **Figures 2A,G**) but bell-shaped inspiratory inward currents became very irregular due to TRH-induced FOCs, which made the amplitude and area incomparable with that in the absence of TRH (**Figure 2H**). Additionally, TRH significantly increased the frequency, peak amplitude and area of the hypoglossal inspiratory bursts (Rekling et al., 1996; Hou et al., 2012). The effects of TRH usually disappeared in about 5 min upon wash, and a second application of TRH at 10–20 min interval caused comparable responses.

### Tetrodotoxin (TTX) Inhibited the TRH-Induced Excitatory Slow Inward Current and Prevented the Oscillatory Pattern

**FIGURE 3:**
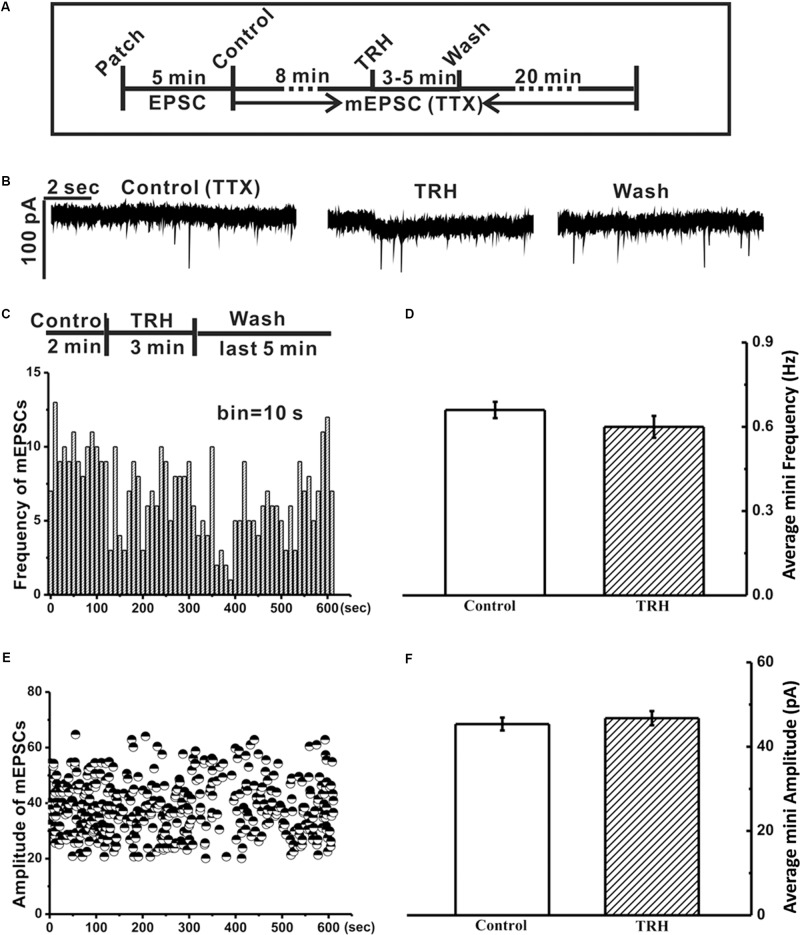
Tetrodotoxin (TTX) inhibited the TRH-induced excitatory slow inward current and prevented generation of FOCs in IA-AVPNs. **(A)** A time axis in this experimental protocol. After TTX was applied for 8 min, TRH was globally applied into the bath for 3–5 min, followed by a 20 min wash with ACSF including TTX. **(B)** Representative mEPSCs recorded under control (left panel), during application of TRH (middle panel), and after 20 min wash (right panel) from an IA-AVPN. **(C,E)** Frequency histogram **(C)** and running amplitude of mEPSCs **(E)** in a representative IA-AVPN, showing TRH caused little alteration in both frequency and amplitude of mEPSCs. **(D,F)** Summarized data of the frequency **(D)** and amplitude **(F)** of mEPSCs in average in IA-AVPNs (*n* = 5).

In five of the twelve IA-AVPNs examined above, TRH was also applied in the presence of TTX (1 μM) (**Figure 3A**). TTX abolished hypoglossal respiratory bursts and the phasic EPSCs during inspiratory bursts in IA-AVPNs, and significantly inhibited tonic EPSCs in both the frequency (from 6.9 ± 2.2 to 0.7 ± 0.03 Hz; *P* < 0.05, *n* = 5; pared Student’s *t*-test) and the amplitude (from 61.1 ± 7.3 to 43.7 ± 1.5 pA; *P* < 0.05, *n* = 5; pared Student’s *t*-test). Under this condition TRH did not cause any change of miniature EPSCs (mEPSCs), neither in the frequency nor in the amplitude (**Figures 3B–F**), and did not cause FOCs in any of these five IA-AVPNs (**Figure 3B**). The TRH-induced excitatory slow inward current was significantly inhibited (**Figure 3B**), from a control of 61.9 ± 16.2 pA to 19.0 ± 7.2 pA in the presence of TTX (*P* < 0.05; *n* = 5). All the mEPSCs were abolished by CNQX (50 μM) at the end of the experiments.

### The TRH-Induced Excitatory Slow Inward Current and the Oscillatory Pattern (OP) in the IA-AVPNs Were Largely Independent of Their Inputs From Chemical Synapses

**FIGURE 4:**
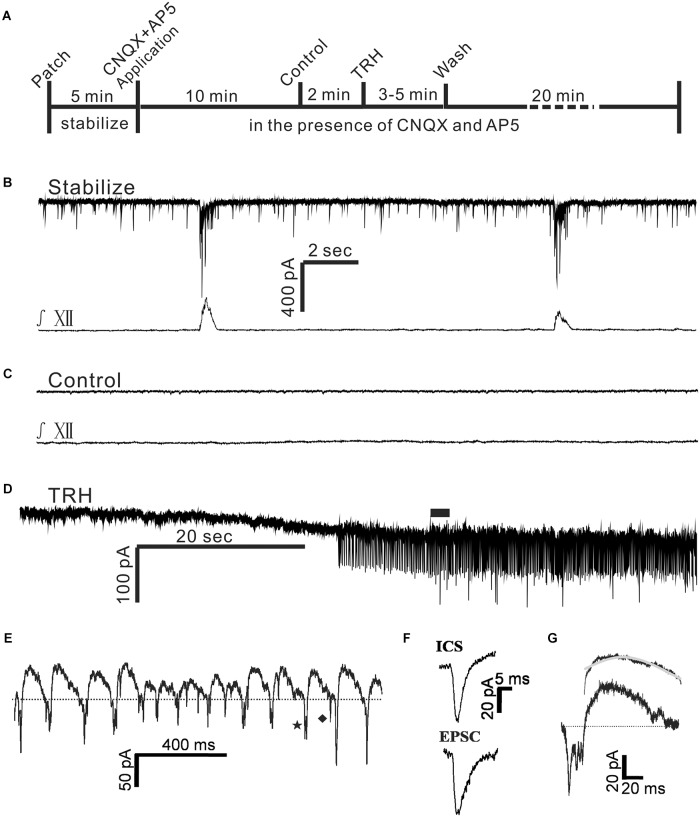
TRH induced an excitatory slow inward current and oscillatory pattern (OP) in IA-AVPNs in the pre-incubation of CNQX and AP5. **(A)** A time axis in this experimental protocol. TRH was globally applied to the patched neuron for 3–5 min after control recording in the presence of CNQX and AP5, followed by a 20 min wash with ACSF including CNQX and AP5. **(B,C)** A representative stabilized recording trace of IA-AVPNs before **(B)** and during bath application of CNQX and AP5 **(C)**. **(D)** TRH induced the excitatory slow inward current and OPs in the presence of CNQX and AP5. **(E)** OPs indicated in **(D)** by the filled bar. Dashed line shows the slower outward phase superimposed by inward current spikelets (ICSs, a typical one indicated by ♦) and the rapid inward phase of FOCs coincident with single or multiple phase-locked ICSs (a typical one indicated by ★). **(F)** Comparison of the kinetics of the ICSs with the EPSCs, showing that the EPSCs had a longer decay time. **(G)** The time course of an oscillatory cycle, showing a rapid inward phase coincident with multiple ICSs and a slower outward phase. The bi-exponential outward phase averaged from 120 oscillatory cycles and the fitted curve (gray) is shown on the top.

**FIGURE 5:**
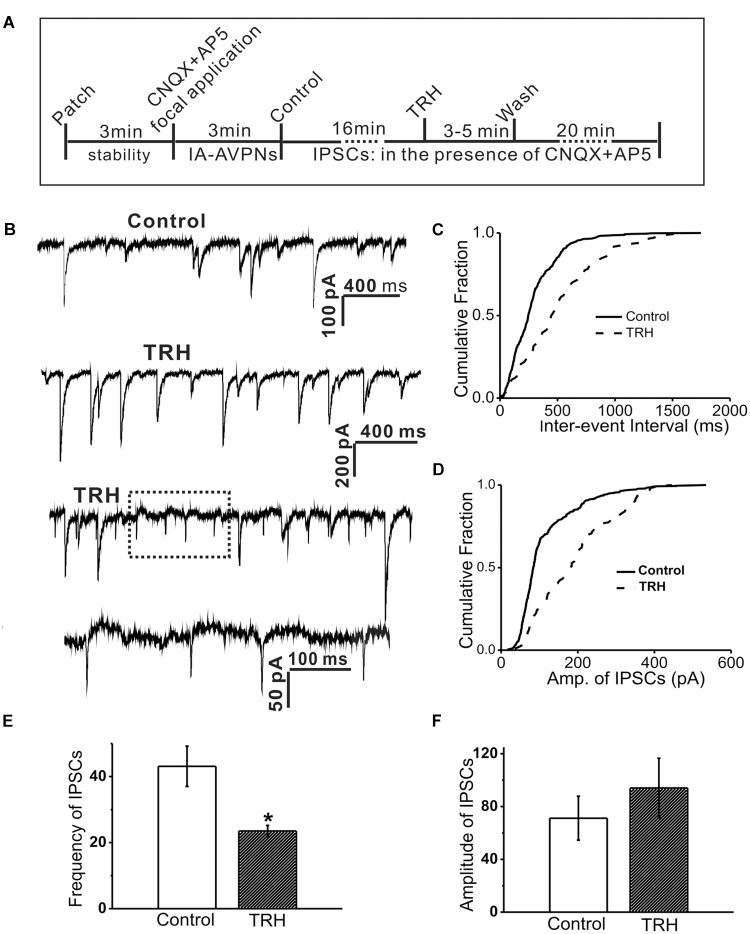
TRH inhibited the frequency and amplitude of spontaneous inhibitory postsynaptic currents (sIPSCs) in IA-AVPNs and simultaneously induced OPs. **(A)** A time axis in this experimental protocol. After IA-AVPN was identified by focal application of CNQX and AP5, the baseline control was recorded in the bath application of CNQX and AP5. TRH was then globally applied to the patched neuron for 3–5 min, followed by a 20 min wash with ACSF including CNQX and AP5. **(B)** A representative recording of control (the first trace) and during application of TRH (the second and third trace), showing that TRH caused an initial increase in the frequency and amplitude (the second trace), and then decreased the frequency and amplitude accompanied by the occurrence of OPs (the third trace). The dashed box in the third trace is shown in an enlarged time scale (the fourth trace). The scale bars in the first trace also applicable to the third trace. **(C,D)** Cumulative plots of IPSCs intervals **(C)** and amplitude **(D)** of IPSCs during control (solid line) and the period of TRH-induced OPs (dashed line; plotted from the same IA-AVPN in **B**). Note that TRH induced a rightward shift in both the inter-event interval and the amplitude distribution curve, indicating decreases in the frequency and increases in the amplitude, respectively. **(E,F)** Summarized data for the frequency **(E)** and amplitude **(F)** of IPSCs in average (*n* = 4). ^∗^*P* < 0.05, Student’s paired *t-*test.

Tetrodotoxin blocked the TRH-induced enhancement of tonic EPSCs, significantly reduced the amplitude of the excitatory slow inward current and prevented the FOCs. These results raised such possibilities as in the absence of TTX TRH-induced enhancement of EPSCs might be critical in the generation of FOCs and might also contribute to the significantly larger excitatory slow inward current via summation. To test these possibilities, TRH was applied to IA-AVPNs pre-exposed to CNQX (50 μM) and AP_5_ (50 μM) (**Figure 4A**). CNQX and AP5 blocked all the EPSCs and abolished hypoglossal respiratory bursts (**Figures 4B,C**). Under this condition TRH (100 nM) caused an excitatory slow inward current of 54.4 ± 11.9 pA (*n* = 36), which was not significantly different from that obtained in the twelve IA-AVPNs unexposed to CNQX and AP5 (*P >* 0.05; independent Student’s *t*-test). Interestingly, in all these 36 IA-AVPNs pre-incubation of CNQX and AP5, TRH still exclusively triggered the FOCs (**Figures 4D,E**). Moreover, the rapid inward phase of each oscillatory cycle was still accompanied by single or multiple phase-locked ICSs, and the slower outward phase was also superimposed by sporadically occurring ICSs (**Figure 4E**). FOCs and ICSs formed the OP. Noteworthily, our finding showed that TRH caused an initial increase (three neurons, as is exemplified in **Figure 5B**) or no changes (one neuron, data not shown) in the frequency and/or amplitude of sIPSCs (**Figure 5A**). However, TRH caused progressive declination of sIPSCs in the frequency and amplitude with its prolonged application, which was accompanied by the occurrence of OPs in all the examined neurons (**Figures 5B–F**). TRH caused no changes in the frequency and amplitude of mIPSCs (data not shown). In order to investigate the effect of IPSCs in the generation of OP, in 13 of these 36 IA-AVPNs, picrotoxin (40 μM) and strychnine (1 μM) were also added into the bath to block sIPSCs before TRH application. Importantly, neither the cycle length nor the amplitude of the TRH-evoked oscillatory currents in these 13 neurones was significantly different from those obtained in IA-AVPNs untreated with picrotoxin and strychnine (*n* = 23). The data were thus pooled together when analyzed. These results demonstrated that the inputs from the chemical synapses were less likely to be involved in the generation of TRH-induced OP and suggest that the summation of the TRH-enhanced tonic EPSCs might contribute little, if any, to the TRH-induced excitatory slow inward current.

In the presence of CNQX and AP_5_ both the cycle length and the amplitude of the TRH-evoked FOCs were quite consistent in the individual neurones. Among different neurones, the frequency ranged from 3.8 to 9.5 Hz (6.4 ± 0.2 Hz; *n* = 36), and the amplitude ran from 20 to 50 pA (meanly 4 20 to 50 pA (meanly 40.4 ± 2.7 pA; *n* = 36). The average amplitude of the TRH-induced FOCs in the IA-AVPNs pre-exposed to CNQX and AP_5_ (*n* = 36) was not significantly different from that in those unexposed to CNQX and AP_5_ (*n* = 12; *P* > 0.05). Furthermore, the rapid inward phase of the FOCs was usually accompanied by one to four ICSs, of which the amplitude was relatively consistent in individual neurons but ranged from 20 to 120 pA (46.5 ± 2.3 pA; *n* = 36) among different neurones. Compared with the tonic EPSCs during control recording, ICSs had a comparable rise time (1.3 ± 0.1 ms for ICSs, *n* = 12; 1.4 ± 0.2 ms for EPSCs, *n* = 6; *P* > 0.05, independent Student’s *t*-test), and a significantly shorter decay time (2.4 ± 0.1 ms for ICSs, *n* = 12; 4.9 ± 0.4 ms for EPSCs, *n* = 6; *P* < 0.05, independent Student’s *t*-test). A comparison was made by average of the difference in the kinetics of ICSs and tonic EPSCs (**Figure 4F**). The outward phase of the FOCs presented a bi-exponential time course, as indicated in the inset to the top of **Figure 4G**, in which a comparison was made of the average (*n* = 120) and the bi-exponentially fitted outward currents (gray). Properties of EPSCs in the absence of TRH during the inspiratory intervals and the inward phase of FOCs as well as ICSs induced by TRH in the presence of CNQX and AP5 were compared in **Table 1**.

**Table 1:** Comparision of properties of FOCs, ICSs and glutamatergic EPSCs in IA-AVPNs in the presence of CNQX, AP5, strychnine and picrotoxin.

	Amplitude (pA)	Rise time (ms)	Decay time (ms)	Frequency (Hz)	*n*
EPSCs	39.5 ± 1.3	1.4 ± 0.2	4.9 ± 0.4	6.2 ± 1.2 Hz	6
ICSs	41.5 ± 4.3	1.3 ± 0.1	2.4 ± 0.1^∗^	6.9 ± 3.2 Hz	12
FOCs	41.9 ± 3.7	2.07 ± 0.3^$^	3.22 ± 0.473	4.7 ± 0.3 Hz^$^	12

*^∗^P < 0.05 from EPSCs, ^$^P < 0.05 from EPSCs or ICSs, ☆P < 0.05 from ICSs.*

In the majority (21 out of 36) of IA-AVPNs to which TRH was applied in the pre-incubation of CNQX and AP5, QX-314 (2 mM) was included in the pipette solution to block activation of voltage-dependent sodium currents and *I*_h_. The cycle length and amplitude of the TRH-evoked FOCs, as well as the amplitude of the TRH-induced excitatory slow inward current in these neurones, was not significantly different from those obtained IA-AVPNs without intracellular QX-314 (values not shown). These data were thus pooled together when analyzed. The results suggested that the blockade of the voltage-dependent sodium channels from the inside of individual neurones under recording cannot prevent the occurrence of the FOCs as well as the concurrent ICSs, and has little effect on the excitatory slow inward current. Thus, it is likely that TRH-induced OP in IA-AVPNs are synchronized group activity, other than the activity of those under recording.

### TRH-Induced OPs Were Insensitive to Membrane Potential

**FIGURE 6:**
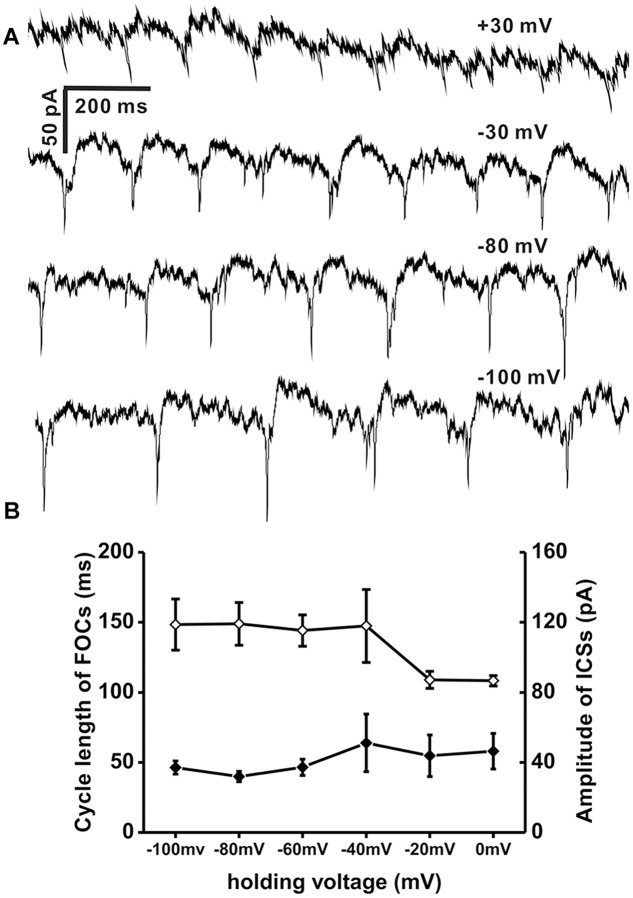
OPs induced by TRH were insensitive to membrane potential. **(A)** A representative recording of OPs recorded at four different levels of holding voltage (values showed above each recording trace) of the same IA-AVPN in the presence of CNQX, AP5, strychnine and picrotoxion, showing that insensitivity of OP cycle length as well as amplitude in ICSs to membrane potential. Pipette solution included QX314 (2 mM). **(B)** Average plots of cycle length of FOCs (♢; left vertical scale; *n* = 4) and amplitude of ICSs (♦; right vertical scale, *n* = 4). QX314 was put into the electrodes in the process of recording.

To test if TRH-induced FOCs as well as the coincident ICSs in IA-AVPNs were network-based activities, holding potential was changed under voltage-clamp recording. In the four IA-AVPNs examined from four slices, once the slow inward current evoked by TRH was in a steady level, the membrane potential was shifted from −100 mV to +30 mV. The cycle length of FOCs and the amplitude of ICSs were both unaltered at four different levels of membrane potentials (*n* = 4, **Figure 6**). Moreover, OPs couldn’t be reversed by the very positive commanded potential of +30 mV in the presence of CNQX, AP5, picrotoxin and strychnine. In this experiment, the pipette solution contained QX-314. Similar voltage shifts didn’t induce OPs in the absence of TRH (data not shown, *n* = 8).

### The TRH-Induced OP Were Prevented by Gap Junction Blocker Carbenoxolone (CBX)

**FIGURE 7:**
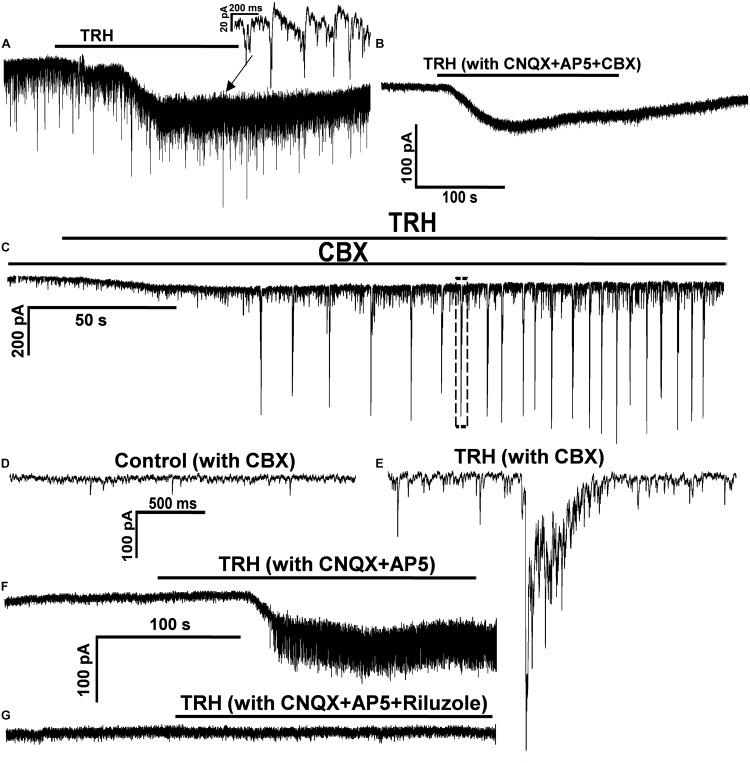
CBX prevented the TRH-evoked oscillatory pattern, and TRH enhanced the EPSCs in the presence of CBX. **(A)** In a representative IA-AVPNs under normal perfusion TRH caused an excitatory slow inward current and evoked OPs. OPs was also evoked in this neurone in the pre-incubation of CNQX and AP5 (not shown). **(B)** In the same neurone in **(A)** CBX prevented OPs without altering the excitatory slow inward current. **(C)** In the presence of CBX, TRH increased the frequency and the amplitude of the tonic EPSCs, and evoked rhythmic bursts of the EPSCs that were coincident with the restored hypoglossal bursts (not shown). **(D,E)** The EPSCs captured from **(C)** before **(D)** and during (**E**: from the dashed box in **C**) TRH application are shown in an enlarged scale. **(F,G)** In a representative IA-AVPNs the TRH-induced OPs and the excitatory slow inward current **(F)** were completely blocked by pre-incubation of 20 μM riluzole **(G)**.

Since inspiratory motor neurones in the NA have been reported to be gap junction coupled (Rekling and Feldman, 1997), TRH-induced FOCs as well as the coincident ICSs in IA-AVPNs might be related to gap junction. To test this possibility CBX (100 μM) was added in the bath after the first TRH application, both in the normally perfused IA-AVPNs (*n* = 5) and in those pre-exposed to CNQX and AP_5_ (*n* = 7). Under both circumstances, 30 min presence of CBX prevented completely the occurrence of the OPs during a subsequent TRH application. However, CBX did not significantly alter the amplitude of the TRH-induced excitatory slow inward current, neither in the normally perfused IA-AVPNs (63.6 ± 17.9 before vs. 56.8 ± 19.3 pA after CBX; *P* > 0.05; *n* = 5) nor in the IA-AVPNs pre-exposed to CNQX and AP_5_ (55.8 ± 20.9 before vs. 56.6 ± 26.1 pA after CBX; *P* > 0.05; *n* = 7). A comparison was made of the TRH-induced excitatory slow inward current in a representative IA-AVPN in the absence and in the presence of CBX (**Figures 7A,B**)

In the normally perfused IA-AVPNs, CBX application inhibited gradually, and abolished finally the hypoglossal inspiratory bursts in a 3–60 min period, and also eventually abolished the bursting EPSCs. TRH was usually applied within the initial 10 min after the disappearance of the bursting EPSCs, under which TRH increased the frequency of the tonic EPSCs from 1.2 ± 0.3 to 5.6 ± 1.4 Hz (*P* < 0.05; *n* = 5), and also increased the amplitude from 32.2 ± 2.4 to 56.1 ± 4.4 pA (*P* < 0.05; *n* = 5). In three of these five IA-AVPNs, TRH caused rhythmic bursting of EPSCs (**Figures 7C–E**), which were coincident with the temporarily restored hypoglossal inspiratory bursts (not shown). At the end of the experiment, EPSCs induced by TRH in the presence of CBX were abolished by CNQX and AP5. These results indicated that TRH is capable of enhancing the EPSCs in IA-AVPNs, and that in the TRH-application experiments performed in the normally perfused IA-AVPNs without CBX, TRH-enhanced EPSCs include both those caused by enhanced glutamate release and those of ICSs coincident with the FOCs.

Additionally, the tonic EPSCs were slowly decreased in frequency and amplitude during CBX application. Since CBX was applied before the second application of TRH at varied time lengths, the CBX-induced gradual changes of tonic EPSCs were not statistically compared.

### Riluzole Decreased the Amplitude of TRH-Induced Excitatory Slow Inward Current and Prevented OPs

Since the summation of TRH-enhanced EPSCs contributed little to the TRH-induced excitatory slow inward current, its inhibition by TTX might be mediated by a direct postsynaptic mechanism, possibly via blockade of the persistent sodium current (INaP), because the blockade of voltage-gated sodium transient with intracellular QX-314 exerted little effect on this current. To test the mechanism, riluzole (20 μM) was added in the bath after the first TRH application to the IA-AVPNs pre-exposed to CNQX and AP_5_; consequently, riluzole alone caused no change of the baseline current in IA-AVPNs. However, 10 min presence of riluzole inhibited significantly the TRH-induced excitatory slow inward current from 51.1 ± 24.3 pA of control to 19.3 ± 10.6 pA (*P* < 0.05; *n* = 7). In four of seven IA-AVPNs, the excitatory slow inward current was actually abolished by riluzole (**Figures 7F,G**). Interestingly, riluzole also completely prevented the occurrence of OPs in these seven IA-AVPNs (**Figure 7G**). These results suggested that activation of INaP might be involved in the generation of both the excitatory slow inward current and the OPs during TRH application.

Importantly, K_ATP_ inhibitor glybenclamide was reported to block the oscillatory currents triggered by the activation of metabotrophic glutamate receptors in hypoglossal motor neurones (Sharifullina et al., 2005). In the current study, therefore, the effect of glybenclamide (100 μM) was tested on the TRH-induced changes in the IA-AVPNs pre-exposed to CNQX and AP_5_; consequently, glybenclamide had no effect neither on the TRH-evoked OPs nor on the excitatory slow inward current (*n* = 3; data not shown).

### TRH Depolarized IA-AVPNs, Causing Continuous or Synchronized Firing Under Current Clamp, Both in the Absence and in the Pre-incubation of CNQX and AP_5_

**FIGURE 8:**
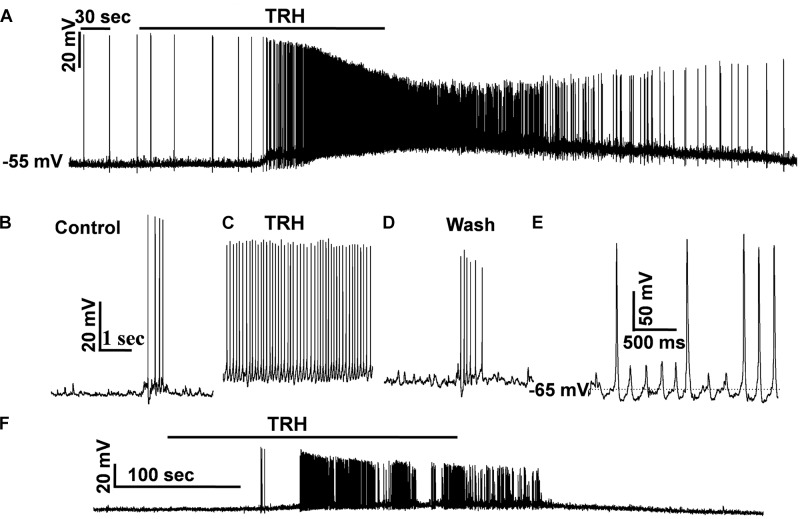
TRH caused continuous firing of the IA-AVPNs and induced increases in firing frequency under current clamp. **(A)** TRH caused depolarization and continuous firing in a representative IA-AVPN. Note the amplitude decrease of action potentials. **(B–D)** Recording of the same IA-AVPN as in **(A)** in an enlarged scale, showing the rhythmic inspiratory depolarization and the superimposed trains of action potentials during control **(B)**, the continuous firing during TRH application **(C)**, and recovery **(D)**. **(E)** The rhythmic oscillatory potentials and the superimposed action potentials when the IA-AVPNs were manually re-polarized during TRH application. **(F)** TRH caused depolarization and continuous firing in a representative IA-AVPN pre-exposed to CNQX and AP_5_.

**FIGURE 9:**
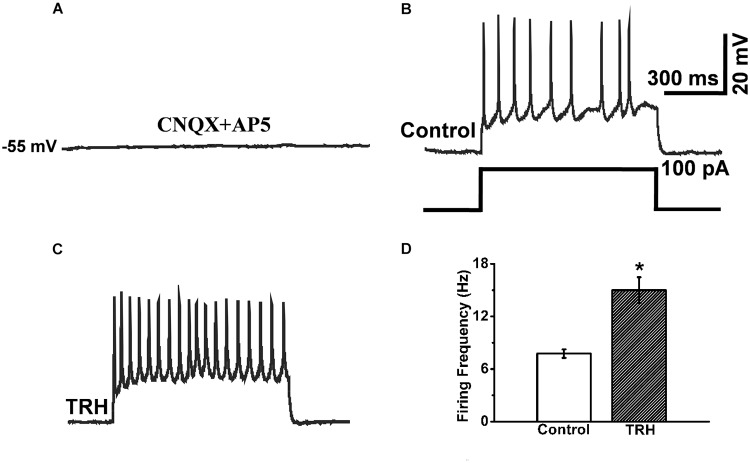
TRH induced increases in instantaneous firing frequency responses to 100 pA depolarizing current. **(A)** In the pre-exposed to CNQX and AP5, an IA-AVPN was silent. **(B)** Injection of 100 pA current induced repetitive instantaneous firing. **(C)** Application of TRH increased the firing frequency in the same neuron in **(B)**. **(D)** Summarized data for the frequency of firing frequency (*n* = 4). ^∗^*P* < 0.05, Student’s paired *t-*test.

To test how TRH affects the firing behavior of IA-AVPNs, TRH was also applied under current clamp, both in normally perfused IA-AVPNs and in those pre-exposed to CNQX and AP5. Under current clamp configuration, the IA-AVPNs showed rhythmic depolarization and action potential firing during inspiratory phase under normal perfusion. Application of 100 nM TRH induced a slowly developing depolarization of 5–25 mV (14.2 ± 3.1 mV; *n* = 5), and the discharge of the IA-AVPNs became continuous, with significantly decreased action potential amplitude (from 70.4 ± 3 mV to 53.1 ± 5 mV; *P* < 0.05, *n* = 5) and significantly increased action potential duration (from 4.7 ± 0.1 to 6.2 ± 0.2 ms; *P* < 0.05, *n* = 5). A typical experiment is exhibited in **Figures 8A–D**. In the pre-incubation of CNQX and AP5, which silenced all the IA-AVPNs (**Figure 9A**), TRH application (100 nM) caused a similar slowly developing depolarization, and exclusively caused continuous discharge (*n* = 5). A typical experiment is exhibited in **Figure 8F**.

In two IA-AVPNs when the membrane potential was depolarized to a level more positive than −30 mV, the discharge ceased both under normally perfused condition and in the pre-incubation of CNQX and AP_5_. The cessation of discharge was obviously because of the inactivation of the voltage-gated sodium channels by over depolarization. When the membrane potential was manually repolarised to a level equal or more negative to the level before TRH application, via injection of a hyperpolarizing current of 10–50 pA, the shape of the action potentials became relatively normal. Under the manually re-polarized condition, moreover, the IA-AVPNs showed fast oscillatory depolarizing potentials, and sporadic action potentials was loaded on the peak of some of these fast oscillatory depolarizing potentials (**Figure 8E**), of which the cycle length was identical to that of FOCs under voltage clamp.

In the pre-incubation of CNQX and AP5, the IA-AVPN was silent (**Figure 9A**), and injection of a square-wave current (100 pA) induced repetitive firing (**Figure 9B**). Under this condition, TRH significantly increased firing frequency from 7.75 ± 0.48 Hz to 15 ± 1.47 Hz (*n* = 4) (**Figures 9C,D**). In the pre-incubation of TTX, TRH didn’t cause any significant depolarization and failed to elicit firing behavior of IA-AVPNs. Under such circumstances, injection of a depolarizing current didn’t trigger oscillatory depolarizing potentials (*n* = 3, data not shown).

## Discussion

The current study exhibits seven main findings:, (1) TRH caused an excitatory slow inward current and the OPs in IA-AVPNs, which were largely independent of their presynaptic inputs; (2) gap junction blocker CBX prevented the TRH-evoked OPs, but had little impact on the excitatory slow inward current; (3) in the presence of CBX, which blocked the hypoglossal respiratory rhythm and inspiratory bursting EPSCs, TRH enhanced the tonic EPSCs and restored the hypoglossal respiratory bursts in some slices; (4) TRH progressively decreased the frequency of the tonic sIPSCs coincident with the occurrence of OPs and had no influence on mIPSCs; (5) TRH-induced OPs were insensitive to membrane potential; (6) TTX and riluzole completely prevented the TRH-evoked OPs in IA-AVPNs, each of them also inhibiting a large proportion of the excitatory slow inward current; and (7) TRH depolarized IA-AVPNs, causing continuous or synchronized discharge under current clamp, both with and without perfusion of CNQX and AP_5_; TRH increased firing frequency responses to input wave-forms.

In general, alterations of a baseline current in the pre-incubation of TTX reveal a postsynaptic mechanism. In IA-AVPNs, an excitatory slow inward current that was inhibited but not completely prevented by TTX during perfusion of TRH, suggesting that TRH had a postsynaptic effect. These results demonstrate that IA-AVPNs have TRH receptors, supporting the previous findings that TRH-containing terminals projected to respiratory motor neurons in NA (Iwase et al., 1992; Sun et al., 1995). In IA-AVPNs, TRH triggered a CBX-sensitive OPs, which was consistent with a previous report that inspiratory motor neurons in NA were coupled by gap junctions (Rekling et al., 2000). The finding also suggests that the generation of the TRH-evoked OPs in IA-AVPNs is a network-based, other than a single-neuron-based, behavior. TRH increased the frequency and the amplitude of the tonic EPSCs in IA-AVPNs in the presence of CBX, and in some slices restored the hypoglossal respiratory bursts and bursting EPSCs that had been abolished by CBX. These results suggest that TRH can have an excitatory stimulation on the glutamatergic inputs of IA-AVPNs, possibly via actions on the pre-terminal sites of the precedent glutamatergic neurons; and that TRH might also be of crucial importance in the respiratory rhythmogenesis via actions on glutamatergic rhythmogenesis neurons.

Thyrotropin-releasing hormone decreased the frequency of sIPSCs accompanied by the occurrence of OPs but didn’t affect mIPSCs, indicating that presynaptic release of inhibitory transmitters was inhibited, in which perhaps two mechanisms involved. One possibility is that the propagation of action potential in the inhibitory neurons preceding the IA-AVPNs determines the presynaptic release of inhibitory transmitters. Once TTX eliminated action potentials mentioned above, TRH had no impact on the release of inhibitory transmitters by just targeting at the terminals of these inhibitory neurons. The other possibility is that OPs induced by TRH affect presynaptic release of inhibitory transmitters. Combined with enhancement of excitatory synaptic inputs induced by TRH, the possible significance of these findings lie in unveiling not only the imbalance of excitatory and inhibitory activities (Moore et al., 2004; Haxhiu et al., 2005) but also the generation of OPs contributes to abnormal activation of IA-AVPNs.

Blockade of chemical synaptic inputs of the IA-AVPNs could not prevent the TRH-evoked OPs. Nicotine and arginine vasopressin applied to the normally perfused IA-AVPNs enhanced the tonic EPSCs and the inspiratory bursting EPSCs, but did not evoke the OPs (Zhou et al., 2013; Yan et al., 2017). These findings suggest that presynaptic inputs are not the underlying cause for the TRH-evoked OP. However, in the normally perfused IA-AVPNs the TRH-evoked OPs had a significantly shorter cycle length during the inspiratory phase compared with that during inspiratory intervals. This finding suggests that the inspiratory-enhanced glutamate release, although cannot trigger the OP alone during inspiratory intervals, might have a promoting effect on this pattern during the inspiratory phase.

Oscillatory currents have been reported to be evoked in the hypoglossal motoneurons by NMDA, nicotine, TBOA (a selective blocker of glutamate transporter), or DHPG (an agonist of group I metabotropic glutamate receptors) (Sharifullina et al., 2008; Cifra et al., 2009). In the current study, TRH-evoked OPs shared some similar properties with the oscillatory currents evoked in the hypoglossal motoneurons, such as blockade by CBX or riluzole, and they also showed some quite different properties, as indicated by the evidence that TRH-evoked OP was independent of the inputs from chemical synapses, sensitive to TTX and resistant to glybenclamide. Whereas the oscillatory currents in the hypoglossal motoneurons evoked by nicotine, DHPG, or TBOA were enhanced by AMPA and blocked by CNQX and K_ATP_ inhibitors (Sharifullina et al., 2005; Cifra et al., 2009). Additionally, both the hypoglossal motoneurons and respiratory rhythmogenesis neurons have been reported to be linked by gap junctions (Rekling and Feldman, 1997; Rekling et al., 2000; Sharifullina et al., 2005). In the current study, TRH caused continuous discharge in IA-AVPNs, while the rhythm of the hypoglossal bursts was maintained. These results suggest that neither in the respiratory rhythmogenesis neurons nor in the hypoglossal motoneurons TRH caused persisted or oscillatory-like excitation, and OPs in IA-AVPNs is a rather specific response of these neurons to TRH. Gap junction couplings existed in the IA-AVPNs are of critical importance in the generation of OPs. This was supported by previous studies that gap junctions were identified between AVPNs in the rostral nucleus ambiguous (Rekling and Feldman, 1997) and were recorded in single IA-AVPNs located in eNA (Chen Y.H. et al., 2007). In the propagation of oscillatory currents, gap junction coupling involves a few HMs (Mazza et al., 1992; Sharifullina et al., 2005). Perhaps gap junctions in IA-AVPNs also involve several neurons in the propagation of TRH-induced OPs. As a limitation, the current study doesn’t use the method of paired patch clamp recording of two IA-AVPNs to clarify it, although CBX inhibits OPs on all the examined single-recorded neurons.

Thyrotropin-releasing hormone increased the bell-shaped inspiratory inward currents in the frequency and induced OPs during inspiratory bursts. In view of the rhythmic activation of inspiratory EPSCs, obviously IA-AVPNs receive synaptic inputs from neuronal network of inspiratory rhythmic generation, e.g., pre-BötC (PBC), or the synaptic inputs mentioned above happen to be the rhythm-generated origin. These are consistent with previous studies that TRH activated respiratory neurons in PBC (Rekling et al., 1996; Ruangkittisakul et al., 2006). In our recent study, gap junction couplings could also be activated by central inspiratory activity during inspiratory phase (Hou et al., unpublished paper). The location of the IA-AVPNs in this study are eNA region, within which the PBC neurons locate. Thus, it makes sense to postulate that gap junctions between IA-AVPNs and PBC neurons might contribute to the generation of OPs during inspiratory phase. Future studies are required to address the question as to whether IA-AVPNs are coupled via gap junctions with PBC neurons. Although we couldn’t provide precise morphological locus of gap junctions at present, it seems that gap junctions here don’t locate at axo-axonal coupling (Schmitz et al., 2001; Sharifullina et al., 2005). This view is supported by the current finding that the cycle length of FOCs is insensitive to membrane potential in a single recorded IA-AVPN in this study. The spikelets induced in hippocampal slice neurons originating from axo-axonal electrical coupling would be inhibited by hyperpolarizing the soma (Schmitz et al., 2001), which was inconsistent with the present findings. Therefore, it is reasonable to infer gap junctions here might locate on somatic-dendritic couplings. Additionally, although we can’t completely rule out the probability that the generation of OPs originates from currents of nearby unclamped neurons, the resistance of input resistance to depolarization or hyper-polarization (from −100 mV to +30 mV) makes it impossible.

Both excitatory slow inward current and OPs induced by TRH were depressed by riluzole (20 μM). This finding indicates that persistent sodium current might play a crucial role in the generation of OPs due to its high sensitivity to riluzole (<10 μM) (Bellingham, 2011). However, riluzole was reported to have a wide-ranging neural effects (Bellingham, 2011), including inhibition of voltage-gated Ca^2+^ current (Bellingham, 2013) and augment of Ca^2+^-dependent potassium current (Beltran-Parrazal and Charles, 2003), etc. It is unlikely that riluzole in this study suppresses presynaptic glutamate release (He et al., 2002; Pace et al., 2007; Rammes et al., 2008; Bellingham, 2013) or blocks ionotropic glutamate receptor postsynaptically (Bellingham, 2013) as OPs evoked by TRH is independent of chemical synapses, although other non-specific effects couldn’t be excluded at present.

It is noted that AVPNs involved in the modulation of lower airway caliber experience developmental changes (Kohn et al., 2009). Especially, as a very crucial factor in the generation of TRH-induced OPs, gap junctions exist not only in inspiratory motoneurons of newborn mouse (Rekling and Feldman, 1997) and neonatal rats (Chen Y.H. et al., 2007), but also in vagal motoneurons in the nucleus ambiguus of adult animals (Lewis, 1994). To our knowledge, gap junction is made up of connexins (Cx). Cx 26 and Cx32, two important components of Cx, were expressed in PBC neurons, and both of them exhibit developmental variations (Solomon et al., 2001). Unfortunately, there is very little information on the expression and developmental changes of gap junctions in IA-AVPNs in the previous literatures. Therefore, it is not known whether OPs in this study could be induced by TRH in adult rats. This issue needs further investigations.

The inspiratory oscillations have been proved in previous studies to play significant roles in facilitating neural output at respiratory motor neurons levels (Huang et al., 1996; Funk and Parkis, 2002; Parkis et al., 2003). In this study, current clamp experiments showed that the IA-AVPNs were overall excited by TRH. Obviously, OPs contributed to increasing firing frequency of IA-AVPNs via the rapid inward phase of FOCs and multiple ICSs. ICSs in this study are likely to be caused by gap-junction communication among neighboring neurons due to their properties and sensitivity to CBX. Increase of such gap-junction communication involve in the synchronization of motor neurons (Kepler et al., 1990; Haxhiu et al., 2005; Sharifullina et al., 2005), although electrical coupling is also demonstrated to reduce motor-neural synchrony in the previous study (Bou-Flores and Berger, 2001). Thus ICSs are likely to play vital roles in the synchronization of IA-AVPNs. Funk et al. documented that the oscillations, especially high frequency oscillations of motor neurons, have high correlations with that of their respective nerves (Funk and Parkis, 2002). Accordingly, it makes reasonable to infer that OPs of IA-AVPNs are possibly synchronized with the oscillations of vagus nerves or airway smooth muscles they innervate. Therefore, OPs of IA-AVPNs might form functional basis for synchronous activation of airway smooth muscles. In addition, because the cycle length of FOCs was insensitive to membrane potentials, the neuronal output was likely to be constrained to the cycle length. Consequently, the emergence of this kind of fast oscillatory depolarizing potentials under current clamp might prevent excessive excitation of IA-AVPNs.

It is not exactly known how TRH induced the excitatory inward current and triggered FOCs in the IA-AVPNs. Based on the current findings, it is likely that TRH first activated of INaP and then opened gap junctions in IA-AVPNs. It seems that INaP contribute to the excitatory slow inward current. The slow outward phase of FOCs might due to the activation of some potassium channels, although TRH could inhibit acid-sensitive TASK channels in locus coeruleus noradrenergic neurons (Ishibashi et al., 2009). Once IA-AVPNs were depolarized to the threshold they fired action potentials, which were then synchronized by opened gap junctions and were detected as OPs in individual IA-AVPNs. The ion channel mechanisms of mediating the rapid inward phase and slow outward phase merit further investigations.

The physiological or pathological significance of the TRH-induced OPs in IA-AVPNs remains unknown. It has been demonstrated that activation of AVPNs by antidiuretic hormone might participate in psychological stress-induced asthma exacerbations in our previous study (Hou et al., 2017). In the previous animal experiment, the central release of TRH after cold stress was seven folds of the control level (Fiedler et al., 2006). Since cold stress is a well-recognized factor in inducing or accelerating asthma, it is likely that the TRH-induced OPs in IA-AVPNs contributes to the genesis or exacerbation of asthma. Network-based OPs mediated by gap junction in this study might be very crucial in the synchronization of IA-AVPNs. Changes in central mechanisms affecting such synchronization might lead to bronchoconstrictive asynchrony. For example, the imbalance of such synchronization might result in a fact that some bronchial branches nearly totally closed but others stay normal when asthma attacks. Alleviation of airway inflammation and airway hyperactivity by inhaled CBX had also been reported (Ram et al., 2009). Thus, ion channel blockers that can inhibit the TRH-induced the slow excitatory currents and block the triggering of the OP might function as promising candidates to prevent and treat asthma.

## Conclusion

In current study, TRH enhances the excitatory inputs, decreases the inhibitory inputs, induces a slow postsynaptic excitatory inward current and triggers an OP mediated by gap junction, all of which contribute to the excitation of IA-AVPNs.

## Author Contributions

LH, LZ, and XZo designed the research. LH, MZ, XZa, ZL, PZ, and DQ performed the research. LH, MZ, XZa, LZ, and XZo analyzed the data. LH wrote the paper. LH, LZ, and XZo revised the paper.

## Conflict of Interest Statement

The authors declare that the research was conducted in the absence of any commercial or financial relationships that could be construed as a potential conflict of interest.

## Abbreviations

4V: 4th ventricle
ACSF: artificial cerebral spinal fluid
AMPA: 2-amino-3-(5-methyl-3-oxo-1,2-oxazol-4-yl)propanoic acid
AP_5_: D-2-amino-5-phosphonovalerate
CBX: carbenoxolone
AVPNs: airway vagal preganglionic neurons
cNA: the compact formation of the nucleus ambiguous
CNQX: 6-cyano-7-nitroquinoxaline-2,3-dione
DHPG: (RS)-3,5-dihydroxyphenylglycine
DMSO: dimethyl sulfoxide
DMV: dorsal motor nucleus of vagus
eNA: the external formation of the nucleus ambiguous
EPSCs: excitatory postsynaptic currents
EPSPs: excitatory postsynaptic potentials
GABA: γ-aminobutyric acid
IA-AVPNs: inspiratory-activated airway vagal preganglionic neurons
ICSs: inward current spikelets
IO: inferior olive
INaP: persistent sodium currents
K_ATP_: ATP-sensitive potassium channels
NA: nucleus ambiguous
NMDA: *N*-methyl-D-aspartate
PBC: pre-Bötzinger complex
PY: pyramidal tract
QX314: lidocaine *N*-ethyl bromide
ROb: raphe obscurus nucleus
NTS: nucleus tractus solitarius
sp5: spinal trigeminal tract
Sp5I: spinal trigeminal nucleus, interpolar part
TBOA: DL-threo-β-benzyloxyaspartate
TRH: thyrotropin-releasing hormone
TTX: tetrodotoxin
XIIN: hypoglossal nucleus
